# Methamphetamine consumption and life-threatening abdominal complications: A case report

**DOI:** 10.1097/MD.0000000000010647

**Published:** 2018-05-04

**Authors:** Xiaojing Zou, Haiyan Huang, Le Yang, Hong Liu, Yongfeng Li, Qin Xia, Shiying Yuan, Shanglong Yao

**Affiliations:** a Department of Critical Care Medicine; b Institute of Anesthesiology and Critical Care Medicine, Union Hospital; c Department of Emergency Internal Medicine, Tongji Hospital; d Department of Gastrointestinal Surgery; e Department of Pathology, Union Hospital, Tongji Medical College, Huazhong University of Science and Technology, Wuhan, P.R. China.

**Keywords:** abdominal pain, hematochezia, methamphetamine, visceral ischemia

## Abstract

**Rationale:**

Methamphetamine (METH) abuse is increasing rapidly all over the world and becoming a significant public health concern in China. However, abdominal complications secondary to METH abuse are usually overlooked. We describe an unusual case of gangrenous cholecystitis and small intestinal ischemia due to METH abuse.

**Patient concerns:**

In this report, a 44-year-old male patient with abdominal pain and hematochezia has a history of crystal meth abuse.

**Diagnosis:**

The patient was diagnosed as septic shock, paralytic ileus, gangrenous cholecystitis, and small intestinal ischemia due to METH abuse based on computed tomography (CT) scan, endoscopy examination, laparotomy, and pathology.

**Interventions:**

Antishock treatment, broad-spectrum antibiotics, and exploratory laparotomy were given.

**Outcomes:**

The patient survived. Six months later, he tolerated oral intake and stopped using crystal METH.

**Lessons:**

Visceral ischemia should be considered if an adult patient with a history of METH abuse is accompanied by abdominal pain and hematochezia.

## 1 Introduction

Methamphetamine (METH), a well-known powerful psychostimulant, exists in 2 stereoisomers, the L- and D-forms. A highly purified D-METH presenting as crystalline form is named as “crystal meth,” which is the most frequently used illicit drugs worldwide.^[1]^ As a potent central nervous system stimulator, METH abuse can result in striking behavioral and cognitive changes and cardiovascular diseases,^[2]^ the abdominal complication of METH consumption is uncommon and conveniently ignored by clinicians. Here, we reported an unusual case of METH-associated visceral ischemia including gangrenous cholecystitis and small intestinal ischemia and explored the possible mechanism. The patient gave consent for these studies and their publication and this report was approved by the Ethics committee of the Union Hospital, Tongji Medical College of Huazhong University of Science and Technology.

## 2 Case presentation

The patient, a 44-year-old man, presented to the emergency department with a 2-day history of abdominal pain and hematochezia. The abdominal pain became worse and had no relieving factors. There was no history of fever, chills, or chest pain. A few hours later, he was then transferred to the intensive care unit because of septic shock, paralytic ileus, and acute respiratory failure. His medical and surgical history was unremarkable except for hypertension controlled with amlodipine. His social history was occasional smoking and intermittent crystal meth use weekly during the last 4 years and a binge of crystal meth abuse 2 days ago.

**Figure 1:**
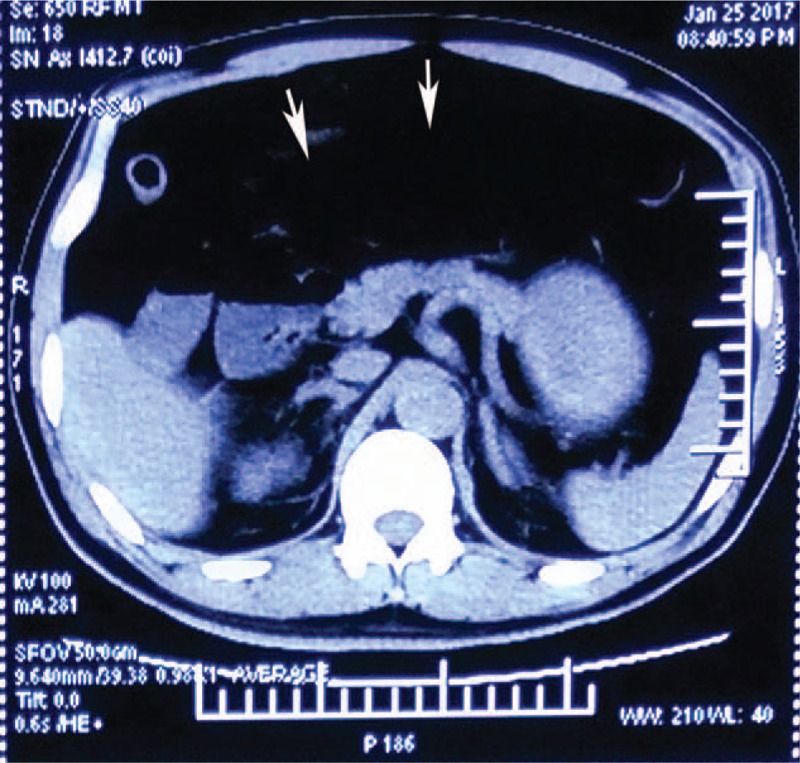
Computed tomography of the abdomen reveals that the small bowel was dilated (the white arrows).

The patient's temperature was 37.1 °C, blood pressure was 86/55 mm Hg, heart rate was 130 beats/minute, respiratory rate was 30 breaths/minute, and saturation of pulse oximetry was 85%. His physical exam was remarkable for tenderness to palpation in the right lower quadrant, a tense and distended abdomen, diminished bowel sounds, and absence of rebound tenderness. The remainder of the physical examination, including a rectal examination, was unremarkable. Initial laboratory analysis revealed that the patient had a white blood cell count of 12.0 × 10^9^ cells/L (its reference value is 3.5 × 10^9^ to 9.5 × 10^9^ cells/L) with 85% neutrophils (its reference value is 40%–75%), hemoglobin of 162 g/L (its reference value is 130–175 g/L), serum procalcitonin of 52.15 ng/mL (its reference value is <0.5 ng/mL), arterial pH of 7.30 (its reference value is 7.35–7.45), PaO_2_/FiO_2_ of 97.8 mm Hg, base deficit of −7.3 mmol/L (its reference value is −2.3 to +2.3 mmol/L), and lactic acid of 9.5 mmol/L (its reference value is <2 mmol/L). The computerized tomography (CT) scan of the abdomen showed dilation of the entire small bowel (Fig. 1).

The patient was treated with fluid resuscitation, broad-spectrum antibiotics, and bowel rest. His physiologic abnormalities were corrected except the diminished bowel sounds after 6 days of treatment.

**Figure 2:**
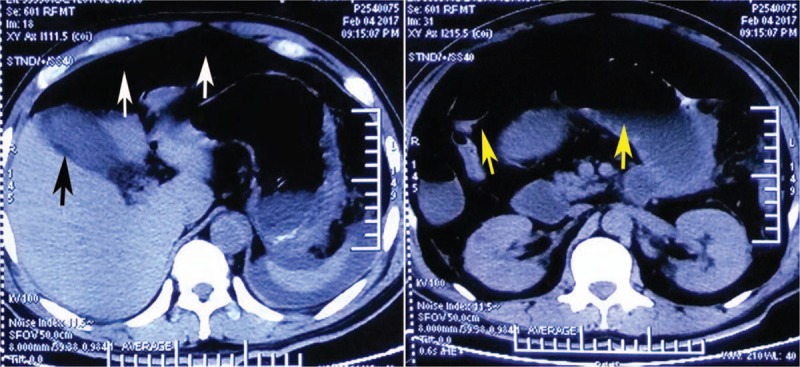
Computed tomography of the abdomen shows cholecystitis (the black arrow), celiac free gases (the white arrows), and gas-fluid levels (the yellow arrows) in the dilated small intestine.

On hospital day 10, however, the patient's abdominal pain presented once again and worsened rapidly. He was in obvious distress. Heart rate was 140 beats/minute, blood pressure was 82/50 mm Hg, and respiratory rate was 35 breaths/minute. The physical exam revealed diffuse rebound tenderness and distended abdomen. An abdominal CT scan was performed repeatedly. The CT scan demonstrated cholecystitis, celiac free gases, gas-fluid levels were found in the dilated small intestine, which suggested perforation of hollow viscus (Fig. 2).

**Figure 3:**
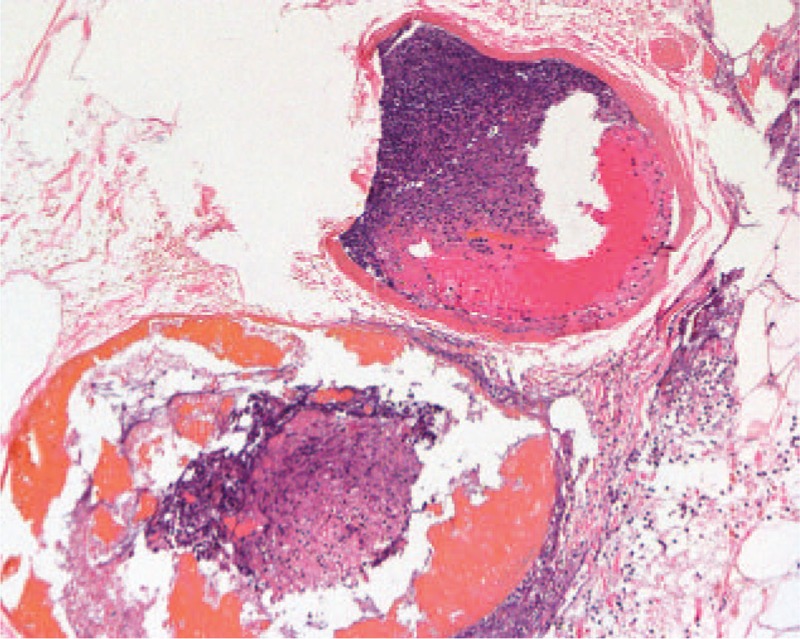
Histopathologic signs of resected cholecyst show mucosal necrosis and small arterial thrombi. Hematoxylin-eosin stain, ×100.

The patient was taken to the operating room immediately for surgical exploration. He underwent an exploratory laparotomy that revealed gangrenous cholecystitis with perforation, edema, and distension of entire small bowel. No perforation, obstruction, or torsion of the bowel was noted. The patient underwent cholecystectomy and bile duct exploration. Histopathologic signs of the cholecyst showed mucosal necrosis and small arterial thrombosis (Fig. 3).

After fluid resuscitation and antibiotics administration, the hemodynamics was stabilized, the abdominal pain and distention were relieved, and the patient was extubated on the second postoperative day.

On the fourth postoperative day, however, the patient developed massive gastrointestinal hemorrhage, hypotension, and worsening abdominal tenderness again.

**Figure 4:**
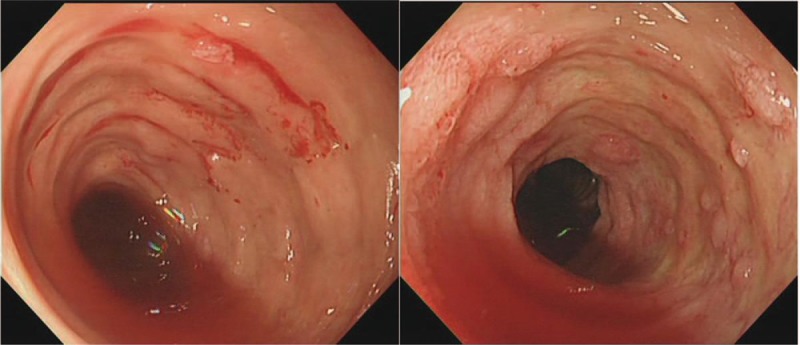
Endoscopic image demonstrates erosion, ulcer, and bleeding in the jejunoileum.

Laboratory analysis revealed an elevated white blood cell count of 16.9 × 10^9^ cells/L, hemoglobin of 89 g/L, and an elevated lactic acid of 5.5 mmol/L. Esophagogastroscopy, colonoscopy, and capsule endoscopy demonstrated edema, bleeding, and multiple ulcerations in the jejunoileal mucosa and normal mucosa in stomach and colon (Fig. 4).

**Figure 5:**
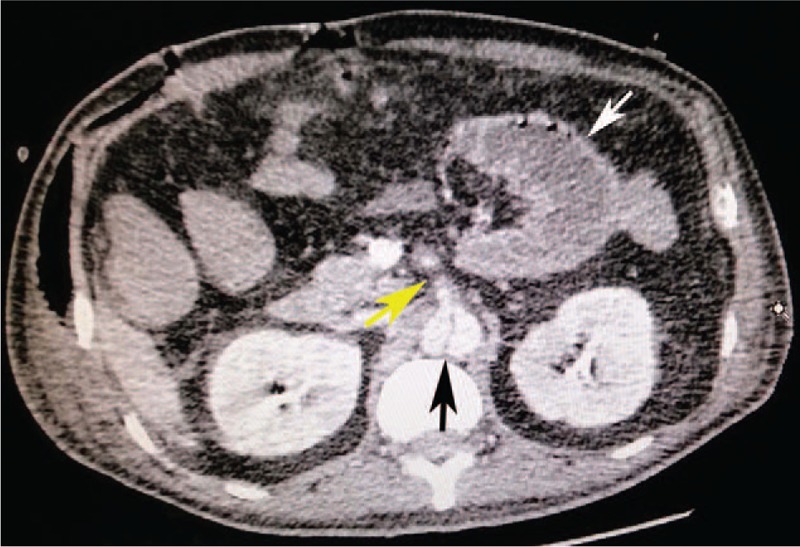
Computed tomography angiogram of the abdomen reveals aortic dissection involving the SMA (the black arrow), nonocclusive thrombosis of the SMA (the yellow arrow), and bowel wall edema (the white arrow).

Furthermore, the patient received angiogram. The findings of abdominal CT angiogram included aortic dissection involving the coeliac artery and the superior mesenteric artery (SMA), nonocclusive thrombosis of the SMA as well as some of its distal branches and bowel wall edema (Fig. 5).

**Figure 6:**
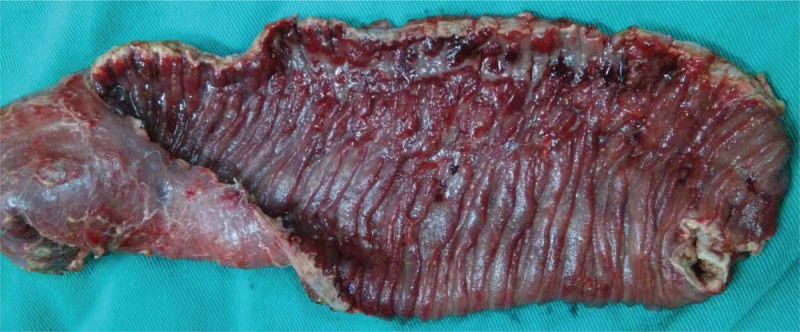
Photograph of the resected specimen of jejunum showing the mucosal ulcer and congestion.

Following fluid resuscitation, the patient was taken to operation room for emergency exploratory laparotomy once again. Intraoperatively, generalized dilatation with edema of small intestine was found, and an isolated ulcer with perforation of 0.7 cm and active hemorrhage also were found in the jejunum, which was 50 cm distal to the ligament of Traitz, and the surrounding jejunum mucosa presented active bleeding. The patient underwent resection of approximately 20 cm of frankly hemorrhagic jejunum with a jejunostomy. Macroscopic appearance of the resected specimen showed the mucosal ulcer and congestion (Fig. 6).

**Figure 7:**
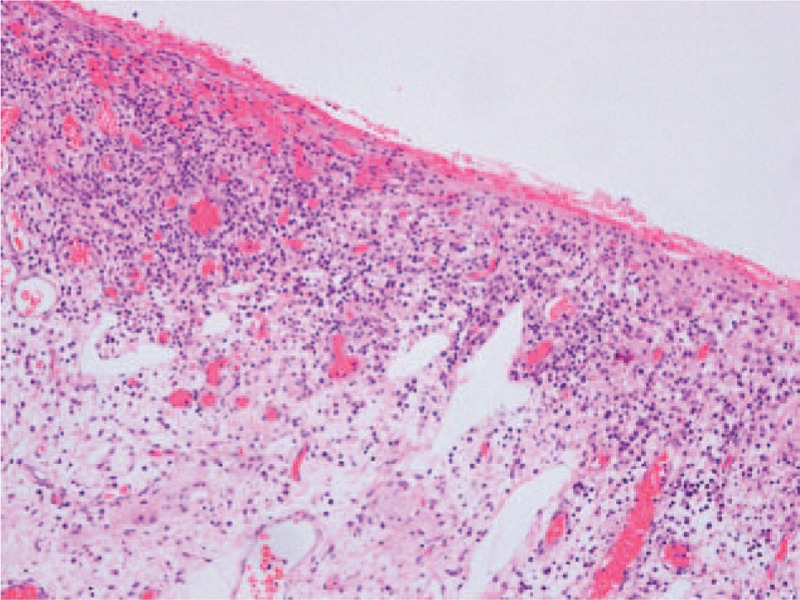
Histopathologic signs of the biopsy sample. Inflammatory cells, mucosal edema, and capillary thrombi are seen within the jejunum. Hematoxylin-eosin stain, ×100.

Histopathologic signs of the resected jejunal specimen demonstrated extensive inflammation, vascular congestion, and multiple ulcerations, one of the ulcerations resulted in small bowel perforation as well as intraperitoneal hemorrhage, inflammatory cell exudates along with submucosal edema (Fig. 7).

Upon the suspicion of extensive inflammation, multiple ulcerations, and nonocclusive thrombosis due to small vessel disease of intestine, workups for autoimmune diseases such as lupus and thrombophilia-including tests for antinuclear antibody, anti-ds DNA, protein C, protein S, antithrombin III, and antiphospholipid antibody were negative.

Moreover, the patient did not have any risk factors or clinical manifestations for small intestine ulcer diseases, such as inflammatory intestinal disease, malignancy, and virus infection. The tests for human immunodeficiency virus, Epstein–Barr virus, and cytomegalovirus were negative.

On postoperative day 2, the hemodynamics was stabilized, lactic acid was normal, no gastrointestinal hemorrhage, and the patient was extubated.

The patient was subsequently discharged. Currently, he tolerates oral intake and he has now stopped using crystal meth or any other recreational drugs.

## 3 Discussion and conclusion

In the present case, all of the events of abdomen include paralytic ileus, gangrenous cholecystitis with perforation, small intestinal ulcerations with perforation, and hemorrhage. Pathology revealed mucosal necrosis and small arterial thrombosis of cholecyst, nonspecific inflammation, and ulcers in appearance throughout the jejunoileum.

During the operation, bilestone, tumor, and biliary obstruction were not found. For acute acalculous gangrenous cholecystitis, we could not overlook the status of the cystic vessel, just like in cases of intestinal ischemia, where we evaluate the mesenteric vessel. We believe that the point of gangrenous ischemia in cholecystitis is the small-sized vessel thrombosis of the gallbladder wall revealed by microscopic examination not the pressure in biliary tract.

Moreover, considering inflammation and multiple ulcers throughout the jejunoileum are found in various diseases, autoimmune diseases, malignancy, and infection were excluded by medical history, manifestation, and tests. It was thought that the patient's small intestinal ulcers were likely ischemic in nature because the CT angiogram indicated aortic dissection involving SMA, nonocclusive thrombosis of the SMA as well as its branches.

Based on above mentioned, the common ground of gangrenous cholecystitis and small intestinal ulcerations of this patient is ischemia.

This 44-year-old patient admitted a 4-year of METH abuse history. Like cocaine, METH can block the reuptake of presynaptic norepinephrine and release the monoamine neurotransmitters dopamine, serotonin, and norepinepherine rapidly and sustainably.^[3–5]^ It accumulates norepinephrine at the postsynaptic level leading to intense arterial vasospasm or vasoconstriction and subsequent ischemia of organ, such as jejunoileum with mucosal and transmural necrosis. Focal tissue ischemia can lead more commonly to ulcerations.^[6,7]^ As the terminal artery, vasospasm of cystic artery induced by METH is easier to cause ischemia of gallbladder.^[8]^

Other physiopathological mechanisms of ischemia include vascular thrombosis and aortic dissection.^[9]^ Although METH is known to cause thrombosis in different sized vessels, such as coronary arteries, cerebral vessels, and aorta,^[10,11]^ so far, thrombosis in small vessel of gallbladder wall and mesenteric artery associated with METH use has been rarely reported.

The cause of our patient's nonocclusive thrombus in the small vessel of gallbladder wall, the SMA as well as some of its distal branches remains unclear. Repeated floods of catecholamines by METH abuse lead to acute accelerated hypertension, tachycardia, and intense vasoconstriction. The arterial walls are now subject to severe shearing forces, which resulted in intimal injury, consequent vessel thrombosis, and aortic dissection.^[12–15]^

The combination of vasospasm, nonocclusive thrombus, and dissection of artery breeds the malperfusion of gallbladder and small intestine. Unfortunately, for this patient, the hypotension, hypovolemia, and norepinephrine infusion due to the septic shock aggravate the low perfusion of gallbladder and small intestine, and ultimately result in gangrenous cholecystitis with perforation, multiple ulcers with perforation, and massive hemorrhage of jejunoileum.

In summary, with the increasing abuse of METH, clinicians should be aware of its potential abdominal complications. Ischemia of celiac viscera such as gallbladder and small intestine should be considered in young adult or middle-aged group with a history of METH abuse who present with abdominal pain and hematochezia.

## Acknowledgments

The authors thank the Natural Science Foundation of Hubei Province (2015CKB744) for the support. The authors also thank MDs Xiaoming Lu, Weikang Zhang, Tao Yin, and Jing Cui for the surgeries.

## Author contributions

**Conceptualization:** Xiaojing Zou, Hong Liu, Shiying Yuan.

**Formal analysis:** Hong Liu.

**Funding acquisition:** Hong Liu.

**Investigation:** Hong Liu, Yongfeng Li, Qin Xia.

**Methodology:** Haiyan Huang, Qin Xia, Shanglong Yao.

**Project administration:** Yongfeng Li, Shanglong Yao.

**Resources:** Haiyan Huang.

**Supervision:** Haiyan Huang, Shiying Yuan, Shanglong Yao.

**Visualization:** Xiaojing Zou.

**Writing – original draft:** Xiaojing Zou, Le Yang.

**Writing – review & editing:** Xiaojing Zou, Le Yang.
